# Paralysis of the Accommodation‒an *a Posteriori* View of Diphtheria
^1^Read at a Meeting of the Bath and Bristol Branch of the British Medical Association, April 30th, 1902.


**Published:** 1902-09

**Authors:** W. M. Beaumont

**Affiliations:** Surgeon to the Bath Eye Infirmary


### PARALYSIS OF THE ACCOMMODATION—AN *A POSTERIORI* VIEW OF DIPHTHERIA.[1]

BY

W. M. BEAUMONT, Esq., M.R.C.S., &c.,

*Surgeon to the Bath Eye Infirmary.*

PARALYSIS of the accommodation is a condition often seen by ophthalmic surgeons following attacks of throat disease of so slight a character that the anginal symptoms have been almost, or, it may have been, quite overlooked. If all these cases are considered diphtheritic, it is probable that more cases of diphtheria are treated by the mothers of England (in consultation with the local druggist) than are treated by the medical profession. The diagnosis of diphtheria has always presented great difficulties, and these difficulties have not been overcome, as was hoped, by the discovery of the Klebs-Loeffler bacillus, for pathologists tell us that it may be present when there is no diphtheria, and that it may be apparently absent when (clinically) there is diphtheria. If we ignore the Klebs-Loeffler bacillus and base our diagnosis on the nerve degeneration, we are possibly in rather smoother water; but, unfortunately, the nerve symptoms are not usually early ones, and it is at the beginning of the attack that the parent or the schoolmaster expects us to give a decisive answer to the question—"Is it a case of diphtheria?" From the ophthalmic surgeon's point of

[1] Read at a Meeting of the Bath and Bristol Branch of the British Medical Association, April 30th, 1902.

view the diagnosis is not difficult if we accept paralysis of the accommodation following ulceration of the throat as proof positive that the disease was diphtheria. Dr. Sidney Martin[1] insists that "the presence of nerve degeneration is the pathological test of diphtheria as a disease. There are other membranous inflammations of the throat due to micro-organisms," he says, "but diphtheria is the only one which leads to nerve degeneration." But paralysis of the accommodation is not universally accepted as conclusive evidence that a preceding anginal attack was diphtheritic. Mr. Jonathan Hutchinson[2] says: "I have seen some cases of this paralysis of accommodation after very slight attacks of diphtheria, and a few in cases of quinsy or pharyngeal abscess where presumably no real diphtheritis had been present." In a foot-note he adds: "Dr. Mansel Sympson in his interesting account of diphtheria as he observed it in Dr. Gee's wards in St. Bartholomew's has the following important observation which fits well with the opinions I have expressed: 'In epidemics, catarrhal diphtheria may occur wherein there is no formation of false membrane whatever. These cases may communicate diphtheria to others and may be followed by paralysis.'" For clinical purposes I think we shall not go far wrong if we consider all cases of anginal inflammation followed by paresis of the accommodation as diphtheria.

From the brief notes of cases of paralysis of the accomodation which I append to this communication, it would seem that slight throat affections are just as liable to be followed by paralysis as severe ones.

That diphtheria is often overlooked is hardly to be wondered at when we consider that so orthodox a symptom as membrane may be absent. Dr. Mansel Sympson, quoted above, is not more strong on this point than Dr. Cobbett and Dr. G. C. Phillips[3]: "During the last few years," they tell us, "the diagnosis of diphtheria has come to be founded more and more on the presence of the Klebs-Loeffler bacillus. So greatly have recent discoveries modified our notions of

[1] *Brit. M. J.*, 1895, ii. 461. [2] *Arch. Surg.*, 1893, iv. 210.

[3] *J. Path. & Bacteriol.*, 1897, iv. 193.

this disease, that we no longer regard the existence of a membrane as a necessary feature, nor indeed as conclusive evidence when present; for we have learned, on the one hand, that diphtheria may attack the throat without causing any membrane, and, on the other, that well-developed membrane may be present in throats that are not affected by that disease, not upon the tonsils only, but also upon the uvula, soft palate, and other parts."

If, for clinical purposes, we consider all cases followed by paralysis of the accommodation as diphtheritic, then the practitioner may have many difficulties in the diagnosis, whilst the ophthalmic surgeon has very few. From his point of view it may be interesting to look backwards and try to find out how often the disease is recognized, and how often it is of so mild a type that the doctor has not been summoned to attend the case. It is so much easier to make a retrospective diagnosis from the vantage-point of an *a posteriori* position : to prophecy when we know.

The accompanying list of thirty-three cases is the last consecutive series that I have seen in hospital practice. The notes are contracted as much as possible, as the point I wish to draw attention to is the frequency of diphtheria being unrecognised.

In these thirty-three cases it will be seen that in only fourteen (42 per cent.) was diphtheria diagnosed: that is, in 58 per cent. it was unrecognised. Fourteen patients were not attended by a doctor at all (52 per cent.), whilst five cases (15 per cent.) were seen by medical men, but were not considered to be diphtheritic. I do not think that the medical profession can be blamed for not calling these five cases diphtheria, for there was no epidemic at the time, and very probably they either were not diphtheria at all, or, if so, of so mild a type that the most careful observer would have doubted its presence. If an examination for the Klebs-Loeffler bacillus had been made and not found, almost certainly they would not have been considered diphtheria; and many of the cases occurred before the value of culture-tests was generally recognised.

| No. | Name. | Sex. | Age. | Date. | History. |
|---|---|---|---|---|---|
| 1 | G. S. | F. | | Jan. 23 | Was at home at Christmas for a week with difficulty in swallowing and swelling of the left side of the throat. She was not seen by a medical man. Convalescent March 6th. |
| 2 | M. F. | F. | 12 | April 21 | Had diphtheria in March, and was ill three weeks. Convalescent in June. |
| 3 | W. M. | M. | 12 | Mar. 31 | Had a sore throat lately, but was not in bed with it, although he was very weak. He could not swallow solids. His mother poulticed his throat, and he did not have a doctor. |
| 4 | W. B. | F. | 12 | Aug. 19 | Was a patient in the Statutory Hospital two months ago for diphtheria. It was a moderately severe attack, and she was ill a month. |
| 5 | T. N. | M. | 12 | Nov. 16 | Sore throat a month ago. Convalescent January 6th. |
| 6 | C. C. | F. | | | Diphtheria a month ago. |
| 7 | F. M. | M. | 12 | Feb. 1 | Diphtheria last July. Convalescent August 21st. |
| 8 | M. B. | F. | 10 | April 14 | Ulcerated throat three weeks ago. In bed fourteen days, but was not attended by a doctor, and no other member of the family was attacked. She has a trace of albumin. Convalescent May 15th. |
| 9 | A. S. | F. | 9 | June 2 | Had a cold and sore throat three weeks ago, and was in bed four days. A chemist gave her some medicine for the cold. The only other child in the family remained well. |
| 10 | R. P. | F. | 8 | Jan. 24 | A sister of No. 9 had diphtheria in December, and a week later she herself was laid up with headache and "something the matter with the water." There was nothing the matter with her throat. |
| 11 | R. C. | F. | 13 | Feb 9 | Was in bed in January with ulcerated sore throat, but only for two days. No diphtheria in the village, but four brothers and sisters had sore throats one after the other, and each was in bed for a day or two. One other child in the village had sore throat with affection of vision. Convalescent March 7th. |
| 12 | A. D. | F. | 10 | Sept. 10 | Influenza five weeks ago, with bad throat; no one else in the house ill, and no doctor attended her. Trace of albumin. Convalescent November 14th. |
| 13 | W. O. | M. | 9 | Dec. 16 | Was attended two months ago for diphtheria. |
| 14 | L. R. | F. | 11 | Feb. 14 | Sore throat December 16th, for which she was attended by a medical man on December 28th, who said it was ulcerated. She was not confined to bed, and she got well in four or five days. |
| 15 | E. R. | F. | 7 | Feb. 12 | Sister of last. Was taken with "mumps," December 12th, followed by diphtheria on December 31st, and sent to the Statutory Hospital, where she remained five weeks, and was treated by injections. |

| | | | | | |
|---|---|---|---|---|---|
| 16 | H. P. | M. | 7 | Mar. 14 | Was attended by a doctor for sore throat in January. In bed four or five days. |
| 17 | D. C. | M. | 7 | Mar. 14 | Had a sore throat and swelling of the neck from February 1st to February 14th. Was in bed ten days, but was not attended by a doctor. |
| 18 | A. W. | F. | 10 | July 4 | Mumps three weeks ago. Was in bed two days; had ulcers of the throat at the same time, but was not attended by a doctor. Convalescent August 29th, but knee-jerks absent. |
| 19 | E. K. | F. | 14 | Nov. 12 | An in-patient at the Royal United Hospital with diphtheria in October. Discharged November 2nd. (The case was considered a doubtful one at the hospital.) Convalescent December 8th. |
| 20 | G. W. | M. | 7 | Nov. 13 | Sore throat five weeks ago; saw a doctor, who did not tell them it was catching. He has three sisters, but none of them were ill. He was in bed seven days. |
| 21 | J. S. | M. | 8 | Jan. 11 | Sore throat on December 1st, and loss of vision since Christmas. Was in bed a week, and was attended by a doctor, but not for diphtheria. Two other boys in the house had it, but are well now. |
| 22 | M. S. | F. | 9 | Aug. 25 | Diphtheria four weeks ago, and paralysis of the accommodation a fortnight ago. |
| 23 | C. B. | F. | 29 | Aug. 27 | Diphtheria a month ago, and loss of vision three days. Was in bed fourteen days. |
| 24 | M. W. | F. | 12 | Nov. 2 | Was in bed with diphtheria in September for seven days. Convalescent November 26th. |
| 25 | C. C. | M. | 13 | Dec. 20 | Had an ulcer of the throat six weeks ago, but was not seen by a doctor during the first week of illness; went to see one the second week. Was not confined to bed at all. Convalescent December 29th. |
| 26 | W. L. | M. | 6 | Jan. 23 | Diphtheria in November. No Klebs-Loeffler bacilli found now. |
| 27 | E. S. | F. | 7 | Jan. 31 | Was in bed four days at Christmas with sore throat, but was not seen by a doctor. A brother had a sore throat a fortnight previous. No diphtheria in district. |
| 28 | A. W. | F. | 8 | Feb. 14 | Was in bed for a week a fortnight ago in consequence of sore throat, epistaxis and headache. |
| 29 | D. H. | F. | 10 | Dec. 16 | Sore throat a month ago; was in bed five days; the glands of the neck were enlarged and there was epistaxis. No doctor was called in. She had nine brothers and sisters in the house, but none of them were ill. Convalescent January 20th. |
| 30 | H. B. | F. | 9 | Feb. 18 | Tonsillitis a few weeks ago; the doctor said it was not diphtheria. Convalescent April 20th. |
| 31 | S. C. | M. | 10 | Oct. 19. | Diphtheria in September. Paralysis of accommodation now. Followed in November by paresis of legs. |
| 32 | A. R. | F. | 8 | July 16 | Diphtheria in June. |
| 33 | A. W. | F. | 10 | July 16 | Diphtheria in June. |

